# Agro-Climato-Edaphic Zonation of Nigeria for a Cassava Cultivar using GIS-Based Analysis of Data from 1961 to 2017

**DOI:** 10.1038/s41598-020-58280-4

**Published:** 2020-01-27

**Authors:** Akinola S. Akinwumiju, Adedeji A. Adelodun, Oluwagbenga I. Orimoogunje

**Affiliations:** 1 Department of Remote Sensing and GIS, School of Earth and Mineral Sciences, The Federal University of Technology, P.M.B. 704, Akure, 340001 Nigeria; 2 Department of Management of Science and Technology Development, Ton Duc Thang University, Ho Chi Minh City, Vietnam; 3 Faculty of Environment and Labour Safety, Ton Duc Thang University, Ho Chi Minh City, Vietnam; 4 Department of Geography, Obafemi Awolowo University, P.M.B. 13, Ile-Ife, 220282 Nigeria

**Keywords:** Plant sciences, Climate sciences, Ecology, Environmental sciences, Mathematics and computing

## Abstract

To investigate the optimal cultivation conditions for cassava cultivar (TMS98/0505) in Nigeria, we employed agro-ecological zoning to delineate the cultivated lands. Using GIS-based multi-criteria analysis, we researched the influence of some meteorological and soil parameters on the clone cultivation. From the multiple-parameter climato-edaphic zoning map, an average yield of 26 t ha^−1^ was estimated. The dry Rainforest and southern Guinea Savanna account for 80% of the favorable zones. However, with irrigation, the cultivar would yield optimally in the northern marginal zones. Further, the significant climatic parameters are sunshine hour (t = 3.292, α = 0.0064) and rainfall (t = 2.100, α = 0.0575). Thus, the potentials of a location for cassava cultivation in Nigeria largely depend on the soil conditions, sunshine hour, and rainfall. Generally, the cassava yield correlates strongly (+0.88) with the suitability map. Considering future climate variability based on the annual rainfall data, we projected an average annual rainfall range of 565–3,193 mm between 2070 and 2099. Likewise, the projected range of daily temperature for 2046–2100 is 24.57–31.94 °C. Consequently, with currently allotted farmlands, Nigeria can double her current cassava production through soil fertility enhancement and irrigation.

## Introduction

Despite its economic importance, cassava has been relegated to marginal lands while preference is given to similar root crops. The low average yield of cassava in Africa can be ascribed to inadequate knowledge of the crop’s inherent benefits, poor on-farm management (such as tilling, spacing, and weeding), and low soil fertility^1–3^. Not until 2005 that the International Institute of Tropical Agriculture (IITA) introduced the cloning of improved cultivars (clones) (such as the cassava mosaic disease- (CMD-) resistant cultivars) to increase the crop’s global yield^2,4^. Since then, significant advancement in cassava yield has been observed. However, the available knowledge on the environmental influence on cassava yield is still insufficient, especially when compared to the incessant researches carried on other cereals and cash crops (such as maize, wheat, cocoa, and oil palm tree)^3,5–11^ globally.

**Figure 1:**
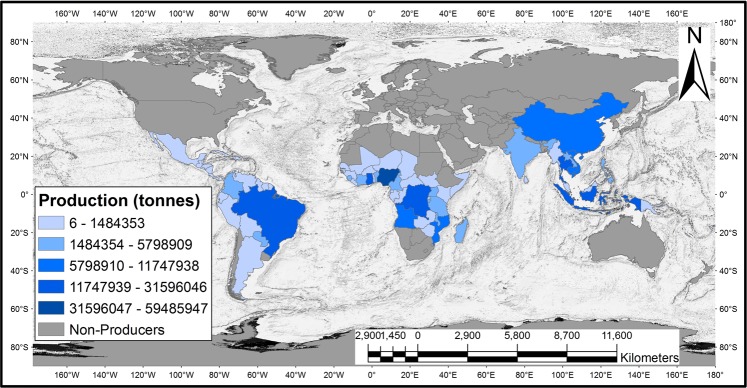
Global cassava production quantity for 2017, indicating the dominance of Nigeria. (FAOSTAT data was processed in the ArcGIS environment).

Nigeria is the largest cassava producer in the world (Fig. 1). Yet, the nation’s cassava cultivation is constrained by diseases, pests, weeds, soil fertility, agronomic factors, and socio-economic factors, which have resulted in the low cassava production^2^. The average cassava yield in Nigeria is 8.76 t ha^−1^, significantly lower than the global average yield of 11.1 t ha^−1^, and much lower than the success stories recorded in India (34.2 t ha^−1^)^12^ and Laos (32.1 t ha^−1^)^13^. Moreover, due to prevailing subsistence agricultural practices solely driven by indigenous knowledge and nature (i.e., lack of irrigation during the dry season) in Africa, crop production in Nigeria is mainly determined by the climatic conditions^14,15^.

It was first reported in 2009 that African farmers will benefit from climate change by 2100^16^. Specifically, cassava cultivation could contribute significantly to climate change adaptation in Africa, based on the crop’s wider tolerance range for moisture availability than other staple crops (such as maize, millet, sorghum, rice, and beans)^10^. Later, it was observed that cassava production will benefit immensely from future climate change in the eastern part of Nigeria^7^. Although, studies have indicated that from 2050 to 2100, the average temperature across Nigeria will increase, with a corresponding decrease in rainfall at the central and southern sub-regions^17–22^. Therefore, cassava cultivation could benefit from the vast lands that will no longer be suitable for cocoa cultivation in the current cocoa belt^23^. Elsewhere, the dominant influences of soil fertility status, fertilizer application, and genotype on cassava yield in the tropics were recently reported^5,24–28^.

The advancement research on cassava breeding at IITA that led to the release of many cultivars (especially the CMD-resistant clones) lacks a comprehensive agro-ecological (A-E) data required to predict the fate of the cultivars under different environmental conditions. Of the five selected CMD-resistant cultivars released in Nigeria, TMS98/0505 is the most widely adopted cultivar, having the highest yields nationwide. However, the variability in the species’ yield across the trial locations (in the tropics) indicates the influence of some environmental factors. Therefore, the necessity of an A-E suitability mapping for cassava production toward an enhanced range yield production in Nigeria prompted this study.

Cassava is a tropical crop sensitive to photoperiod, temperature, and moisture^29–33^. The highest production is expected in the tropics, with temperature and annual rainfall amount ranges of 25–27 °C and 1200–1500 mm, respectively^34^. However, greenhouse and on-farm experiments showed that cassava has a wider tolerance range for temperature (15–35 °C) and rainfall (500–5000 mm)^35^. Likewise, hothouse experiments indicated that the optimum light period for cassava is 12 hours and that longer photoperiod inhibits starch storage capacity^34,36–38^. The on-farm trials have shown that cassava strives in the low-lying areas (altitude < 105 m)^34,37,38^ whereas some cultivars could perform optimally at higher altitudes (≥1500 m)^31,33^.

Outside Nigeria, the A-E zoning for staple crop production has been reported, especially in Ethiopia^6^ and Tibetan Plateau^38^. However, in Nigeria, only the studies to assess the suitability of various geographical zones for the production of wheat and cocoyam have been carried out so far^11,39^. In one of the reports, the minimum variance technique of hierarchical clustering was adopted to group 19 stations in Northern Nigeria based on the A-C potentials for wheat production^11^. Much later, land suitability evaluation to delineate Nigeria into suitability zones for cocoyam production was carried out^39^. The latter study^39^ delineated Nigeria into five zones ranging from unsuitable (in the arid zone) to highly suitable (in the montane region). While A-C zoning emphasizes on climatic conditions^11^, climato-edaphic zoning considers the influences of the climate and soil characteristics on the suitability of a given location for crop production. Aside from the crop-specific A-C zoning, attempts had been made to delineate Nigeria into various A-C zones for staple crop production^14,15^.

The use of Geographic Information System (GIS) for A-C zonation is uncommon in Nigeria. However, both in terms of automated spatial statistical analyses and image analyses, GIS has been reportedly used for A-E zonation^40–44^. Similarly, the delineation of West and Central Africa (WCA) to various A-E zones was done toward understanding the impacts of species richness, on-farm management practices, and environmental constraints^9^. A strong correlation between WCA A-E zones and their respective A-C zones was observed. Also, a south-north decreasing trend of seasonal rainfall was noticed. Zoning techniques have also been employed to upscale simulated yield potentials of crops^45,46^. In current work, we report on the agro-climato-edaphic zonation for cassava production in Nigeria. The research objectives were to (i) model an A-C zonation for cassava production in Nigeria, (ii) map the regions of Nigeria on the basis of the similarity of soil conditions and their suitability potentials for cassava production, (iii) assess the dependence of cassava yield on climate and on soil properties, and (iv) compare the cassava yield trend in Nigeria with those of the leading producing nations in Africa and in the world.

## Materials and Methods

### Study area description

**Figure 2:**
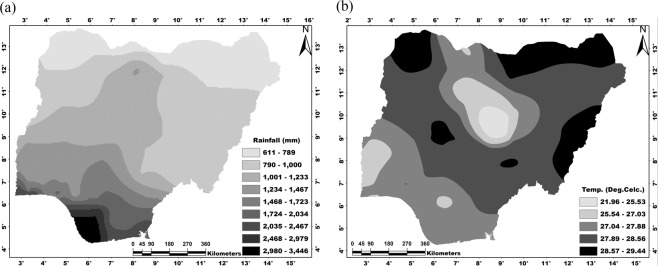
Historical (**a**) annual rainfall amount (1981–2017) and (**b**) daily average temperature (1981–2017). Maps were generated in the ArcGIS environment using CRU data (www.cru.uea.ac.uk/data).

Nigeria lies within latitudes 4°N and 14°N and longitudes 3°E and 14°E. The country has a land area of about 923,769 km^2^, a north-south length of about 1,450 km and west-east breadth of about 800 km. The country has diverse biophysical characteristics and A-E zones. The climate of Nigeria is characterized by strong latitudinal zones which become progressively drier from the coast (in the South) to the hinterland (in the North). There are two seasons in Nigeria: wet and dry, with rainfall as the key climatic variable. Usually, it rains for seven to eight months per year. The total annual rainfall ranges from 3,800 mm at the coast to less than 650 mm at the extreme Northeast (Fig. 2a). Nigeria’s climate is characterized by relatively high temperatures throughout the year. The average annual maximum temperature varies from 35 to 31 °C (in the North and South, respectively), whereas the average annual minimum temperature ranges from 23 to 18 °C (in the South and North, respectively) (Fig. 2b).

The pattern of daily temperature is influenced by altitude while latitudinal variation is evidenced in the south-north rainfall pattern. On the Plateau of Jos and the eastern Highlands, the high altitudes result in temperatures as low as 14 °C. The relative humidity is usually high throughout the year, particularly in the South. In addition, because of the characteristic shorter day and the long night, the south is not favorable for grain crops that require long daylight for their yields.

**Figure 3:**
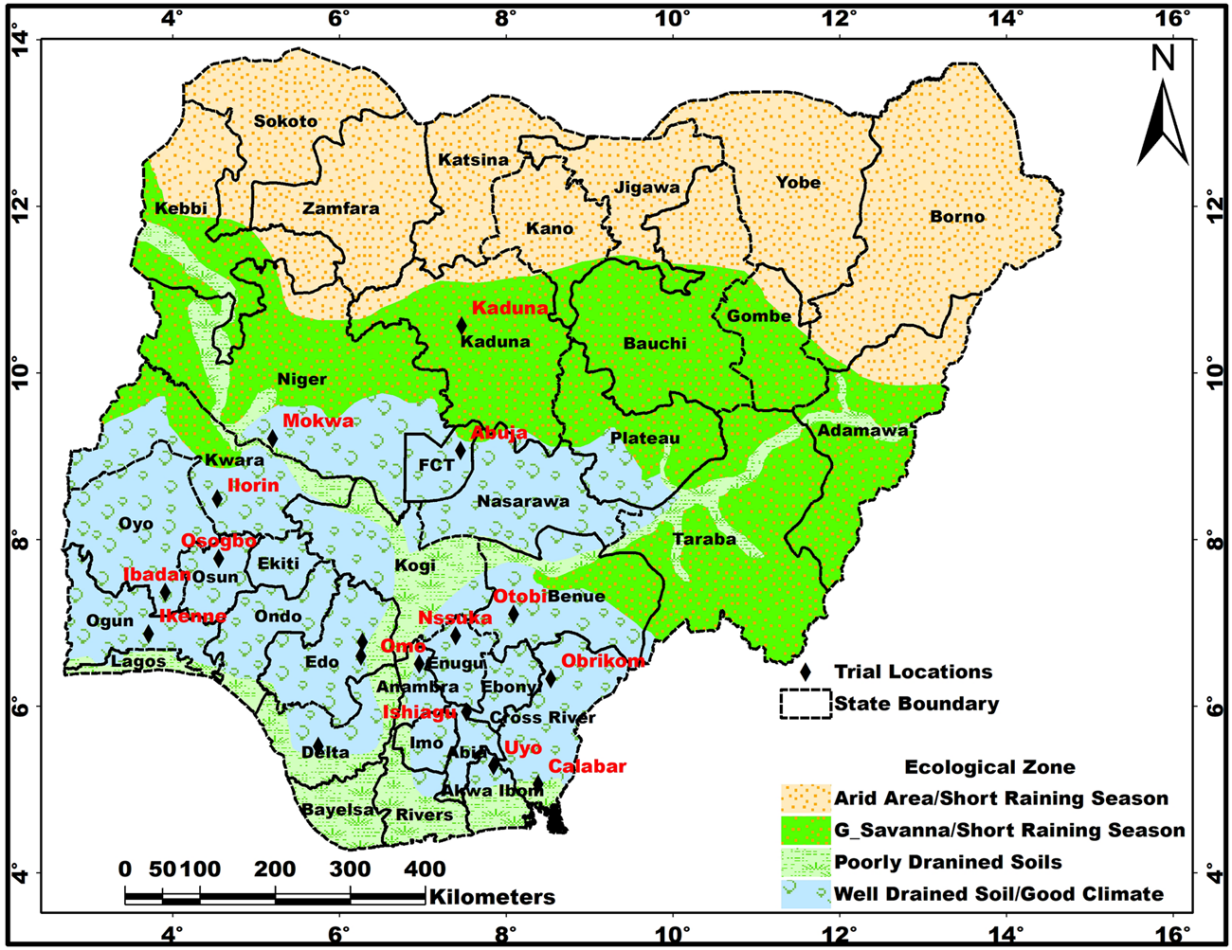
Ecological zones of Nigeria showing the vegetal covers and soil characteristics based on prevailing climatic conditions, vegetation patterns, and cropping systems. This map was generated on ArcGIS platform using rainfall, temperature, and vegetation patterns, with prevailing agricultural practices.

The broad pattern of soil distribution in the country reflects the influence of both climatic conditions and the geological structure. Heavily leached (reddish-brown) and light-to-moderately leached (yellowish-brown) sandy soils dominate in Southern and Northern Nigeria, respectively. The highly weathered soils (prominent in the Southeast) have limited nutrients for crop nourishment. However, soils with high nutrients are abundant in the Northcentral and Southwestern areas of the country, attributed to the underlay rocks (highly mineralized old crystalline basement complex). The vegetal cover of Nigeria (Fig. 3) is characterized by Savanna in the North and Rainforest in the South.

### Data collection and management

We sourced for climatic data from Nigeria Meteorological Agency (NIMET) and 19 other (private and public) institutions in Nigeria. The 32-year rainfall, temperature, and sunshine-hour sub-data were extracted from the Climate Research Unit (CRU) data on Ferex Platform from 61 weather stations across the nations’ six A-E zones. Also, we downloaded the gridded climate research unit (CRU v3.23, monthly at 0.5° horizontal resolution) data (1901–2016) provided by the University of East Anglia. The CRU data was prepared by the Climate Research Unit based on the monthly mean values collated from more than 4,000 weather stations worldwide (www.cru.uea.ac.uk/data)^46^. Elevation data were extracted from SPOT DEM data, sourced from Office of the Surveyor-General of the Federation, Abuja, Nigeria. Cassava yield data and climatic information were obtained from the Cassava Breeding Unit of IITA, Ibadan, Nigeria. Also, the soil map and soil suitability potential map were downloaded from the webpages of the Federal Department of Agricultural Land Resources, Kaduna, Nigeria and Food and Agriculture Organization (FAO), respectively. We clipped the land use map of Nigeria from a gridded land use map of Africa generated from Sentinel 2 in 2016. Total rainfall, daily average temperature, and sunshine hour for the planting season were computed from the recorded climatic parameters. Since moisture availability is a key factor for crop yield performance in the tropics, the duration of the rainy season was also considered as an input variable. Finally, we extracted soil resource information from the soil maps and converted them to quantitative data in the ArcGIS environment. The computed values of the climatic parameters, the elevation data, and the cassava yield data were all converted to point geodatabase in ArcGIS environment. We derived a 32-year average for each climatic parameter extracted from CRU data and were integrated into the point geodatabase. All maps in this study were generated using ArcGIS software (ArcGIS 10.5, ESRI, USA; www.esri.com/en-us/home).

### Analytical procedure

We adopted a weighted overlay algorithm to integrate seven environmental parameters (rainfall, temperature, sunshine hour, length of the wet season, altitude, soil conditions, and land use) in ArcGIS 10.41 environment to generate a suitability zonation map for cassava production in Nigeria. We explored the on-farm trial cassava yield data using a spatial analyst tool on the ArcGIS platform. ArcGIS-based ordinary least square (OLS) was used to model the relationship between cassava yield and environmental parameters (rainfall, temperature, sunshine hour, and altitude). The descriptive statistical analysis of the FAOSTAT cassava yield data using R statistical and Microsoft Excel packages was done^3,5,45,47^. The station-based climate data were subjected to regression analysis whereas the satellite-based gridded climatic data were used for the suitability mapping. For the A-E zoning, we considered the cost of preparing a hectare of farmland (i.e. clearing, procurement of cassava stems, cost of planting, the cost of farm management (such as weeding), and the cost of harvesting as part of the criteria for classifying the soils to different suitability classes. These costs were considered in FAO’s soil suitability classification^48^. Thus, the study area (Nigeria) was delineated into 7 zones viz: zone 1 (*not suitable*), zone 2 (*very marginal*), zone 3 (*marginal*), zone 4 (*moderately suitable*), zone 5 (*suitable*), zone 6 (*very suitable*) and zone 7 (*most suitable*).

### Data derivation and reclassification

To prepare the required inputs for the weighted overlay analysis, the interpolation module was employed to generate raster surfaces from the point-based environmental dataset on climate, altitude, and soil. The raster datasets were reclassified based on predetermined ranges of internal values, informed by data on the phenological responses of cassava to varying ranges of environmental parameters. The adopted class intervals for reclassification of the derived data are presented in Table 1. The derived data were reclassified to conform to weighing scale that ranged from 1 to 9, where 9 and 1 are the highest and lowest suitability rating, respectively. We adopted a weighted overlay analysis module to integrate all the derived data and thus generate an agro-climato-edaphic suitability map that depicts various potential suitability zones for cassava production with varying degrees of prospects and limitations.

**Table 1:** Reclassified input parameters and their internal ratings.

	Rainfall			Temperature			Sunshine Hour			Altitude			Duration of Wet Season			Edaphic factor		
Range	5000–2001	2000–1000	999–500	40–29	28–25	24–11	10–8	7–5	4–3	1500–360	359–150	149–100	10–9	8–7	6–5	15–13	12–10	9–7
Class	3	1	2	3	1	2	1	2	3	1	2	3	1	2	3	1	2	3
Scale Value	7	9	8	5	9	8	9	8	7	9	8	7	9	8	7	9	8	7

### Assigning restrictions and percentage of influence

We introduced land use/land cover data (Table 2) to exclude zones permanently unavailable for cassava cultivation (such as water bodies, human settlements, swamps, canals, gullies, etc).

**Table 2:** Land use/land cover classes and their internal ratings.

^a^LULC type	Class	Scale Value
SWP1, ROC, FP, GUL, MIU, WB, SWP2, RWB, CN, MAU, LP, MA, MF, FFW, SMTF	1	Restricted
SDA	2	1
FA	3	3
MG	4	4
SMRA2, SMRA3, TGP, MTF	5	5
DGBS, GL, RF	6	6
ALV, AD	7	7
SMRA1, DGST, DTWSG	8	8
DSGT, IP, ATCP, RACP, DF, UF	9	9

^a^Acronyms for LULC types are defined as follows:

SWP1: Shrub/Sedge/Graminoid Freshwater Marsh/Swamp, ROC: Rock outcrop, FP: Forest Plantation, GUL: Gullies, MIU: Minor Urban, WB: Natural Waterbodies: Ocean/River/Lake, SWP2: Graminoid/Sedge Freshwater Marsh, RWB: Reservoir, CN: Canal, MAU: Major Urban, LP: Livestock Project, MA: Mining Areas, MF: Mangrove Forest, FFW: Forested Freshwater Swamp, SMTF: Saltmarsh/Tidal Flat, SDA: Sand Dunes/Aeolian, FA: Floodplain Agriculture, MG: Montane grassland, SMRA2: Extensive (grazing, minor row crops) Small Holder Rainfed Agriculture, SMRA3: Extensive Small Holder Rainfed Agriculture with Denuded Areas, TGP: Teak/Gmelina Plantation, MTF: Montane Forest, DGBS: Discontinuous grassland dominated by grasses and bare surface, GL: Grassland, RF: Riparian Forest, ALV: Alluvial, AD: Alluvial Deposit, SMRA1, DGST: Dominantly grasses with discontinuous shrubs and scattered trees, DTWSG: Dominantly trees/woodlands/shrubs with a subdominant grass component, DSGT: Dominantly shrubs and dense grasses with a minor tree component, IP: Irrigation Project, ATCP: Agricultural Tree Crop Plantation, RACP: Rainfed arable Crop Plantation, DF: Disturbed Forest, UF: Undisturbed Forest.

A percentage of influence was assigned to each input parameter based on contribution to the suitability of each location for cassava production in Nigeria (Table 3). Here, two types of influence were considered: (i) the impact of each parameter on the cultivation, survival, and yield of cassava, and (ii) the availability of land for cassava production regardless of its suitability status. Weighting scores were assigned based on greenhouse and on-farm phenological responses of cassava to environmental variables and the influence of land tenure system on crop production^1,2^.

**Table 3:** Weighing scores for the input environmental variables.

S/N	Environmental Variables	Weighting Score (%)
1	Rainfall	20
2	Soil	20
3	Land use/land cover	20
4	Temperature	10
5	Sunshine Hour	10
6	Altitude	10
7	Length of Growing Season	10

## Results

### Suitability zones

**Figure 4:**
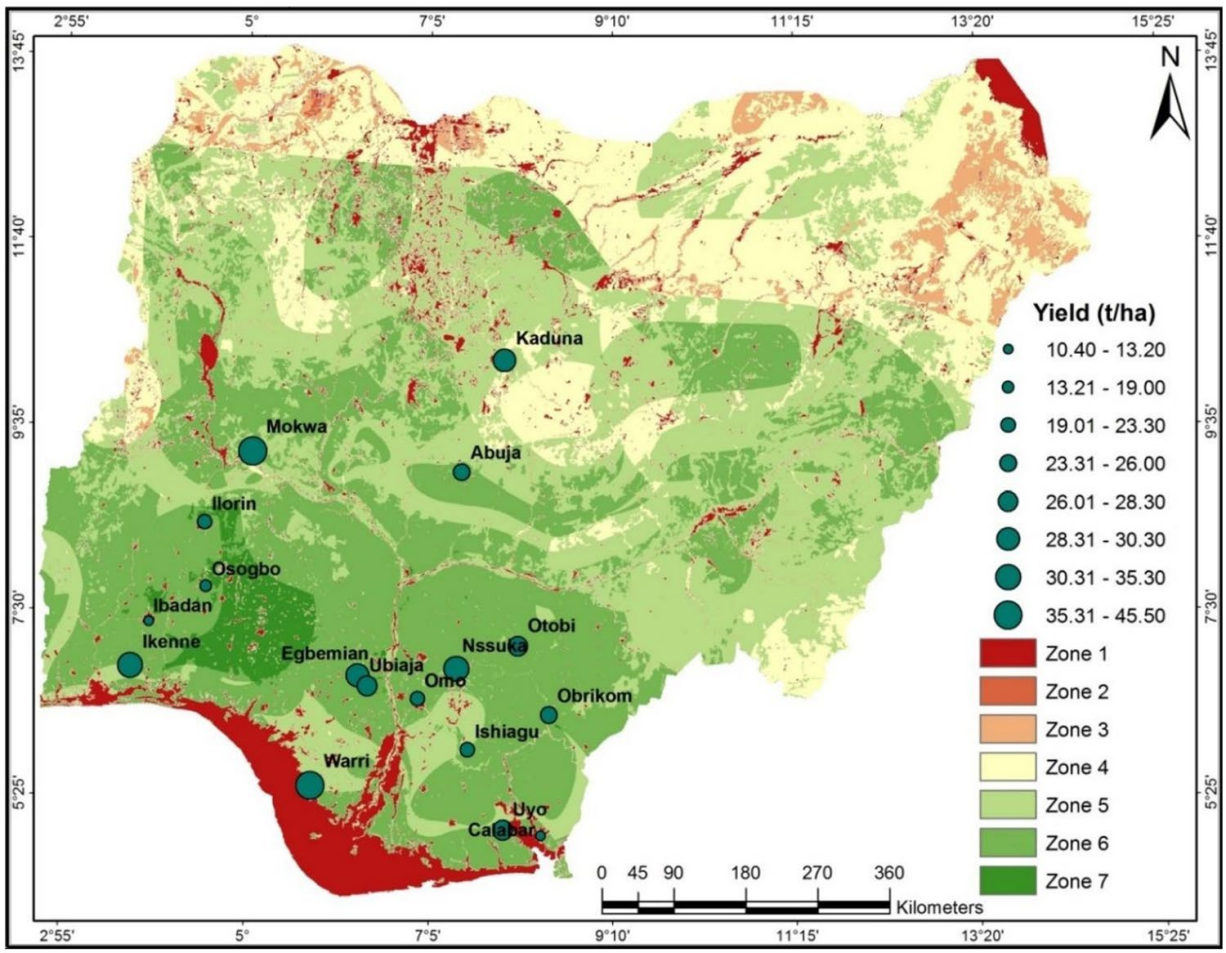
Suitability map of Nigeria for cassava cultivation showing the locations of trial farms and cassava yield. Zone 1: not suitable; Zone 2: very marginally suitable; Zone 3: marginally suitable; Zone 4: moderately suitable; Zone 5: suitable; Zone 6: very suitable; Zone 7: most suitable (Refer to Figure S1). Map was generated using ArcGIS 10.5 software.

Seven suitability zones were delineated using the GIS-aided integration of the seven environmental parameters (Fig. 4 and Table 4). The input raster files, climatic zones, and the edaphic zones (related to the agro-ecological zone map for cassava production) are provided as Supplementary Information (SI1). Zone 1 (*not suitable)* consists of areas unavailable for cassava production (either natural or man-made, such as swamps, urban structural areas, canals, and gullies). Note that the shrinking surface areas of Rivers Niger and Benue have rendered some areas formally inundated available for cultivation in the central part of Nigeria. However, these areas are prone to river flooding during excessive upstream discharge of the major rivers. Zone 2 (*very marginal*) and Zone 3 (*marginal*) are areas not economically suitable for cassava production because the cost of required inputs outweighs the price of the expected yield per hectare. Zone 4 (*moderately suitable)* are areas that require intensive farm management practices (such as major irrigation and soil fertilizer application) to become viable for cassava production. Zone 5 (*suitable*) consists of cultivable areas that require moderate farm management practices (such as soil water loss reduction). In Zone 6 (*very suitable*), cassava cultivation would excel adequately and naturally. Zone 7 (*most suitable)* is the dry rain forest belt and the southern guinea savanna where all the environmental variables are in optimal proportions for cassava production. In Table 4, we report that profitable cassava production could be successfully undertaken on 60,913,264 hectares of land in Nigeria.

**Table 4:** Suitability zonation for cassava farming and land coverage area in Nigeria.

Zone Code	Suitability Zone	Area (ha)	Percentage
1	Not Suitable	8,406,289	9.10
2	Very Marginal	92,377	0.10
3	Marginal	3,390,229	3.70
4	Moderate	19,583,880	21.2
5	Suitable	30,900,040	33.5
6	Very Suitable	28,193,400	30.5
7	Most Suitable	1,819,823	1.97

The ideal number of cassava plants per hectare is 10,000. Based on this, cassava productions are profitable in high yield potential areas (i.e. Zones 7 and 6) only but uneconomically viable in the low yield potential areas (Zones 2 and 3) (Table 5). In our estimation, we have assumed cultivation devoid of fertilizer application and irrigation. In addition, cassava cultivation could be made profitable in the medium yield potential areas (Zones 4 and 5) if rainfall is complimented with irrigation to ensure adequate soil moisture is available within the root zone during the dry season. Nigeria’s climatic and soil conditions, which determine regional suitability for crop production, are complex. Despite the strong correlation between climate and soil, there are differences in their spatio-temporal complexity and variation. In this case, climatic characteristics are less spatially variable but more temporarily complex and unstable^17–19,49^.

**Table 5:** Cassava production cost, fresh root product price, and estimated proceeds in Nigeria (based on current exchange rate $1≡₦306.95 k).

Production Cost		Fresh Cassava Product Price per hectare			
Task	Cost ($)	Yield potential	Price ($)	Suitability zone	Proceed
Farm preparation	488.4	High	977.4	7, 6	244.4
Farm management	244.3	Medium	651.6	4, 5	−81.4
Total	733	Low	325.8	2, 3	−407.2

**Figure 5:**
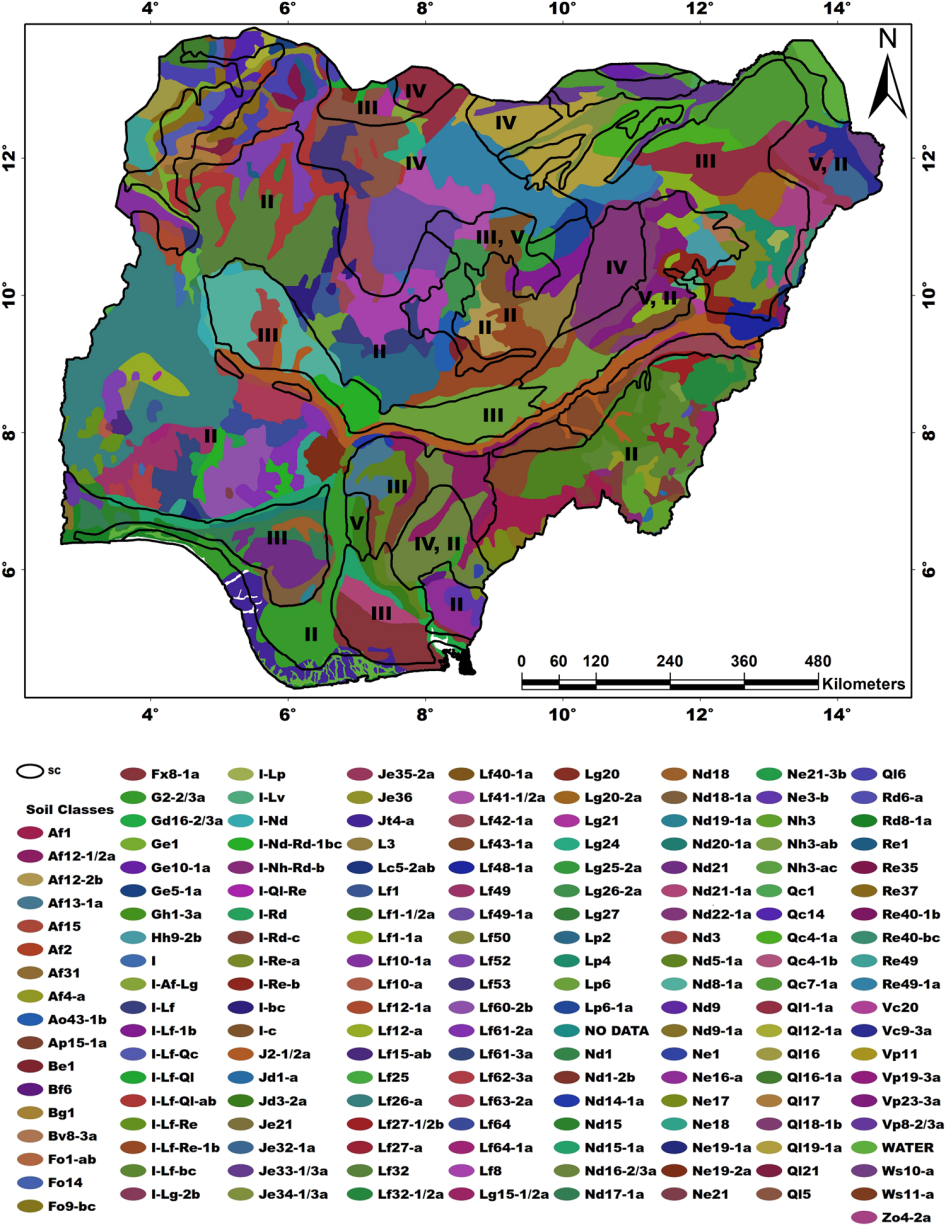
The soil map of Nigeria. The map unit codes indicate the soil associations, texture, terrain characteristics, and suitability classes. The first two letters represent the group and the sub-group, respectively, dominant in each association. The numbers denote the soil texture:1 for coarse, 2 for medium, and 3 for fine. Lastly, the lower-case letters indicate the degree of flatness viz: (**a**) represents flat to gently undulating terrain (0–8%); (**b**) indicates rolling to hilly terrain (9–30%), and c indicates strongly dissected mountainous terrain (>30%). SC color code denotes the suitability classes. Supplementary Information (SI2) provides detailed information on the legend. Map was generated using ArcGIS 10.5 software.

On the other hand, soil characteristics are more spatially and temporarily stable but very complex (Fig. 5). In the southern part of the country where altitude, temperature, and rainfall are very favorable, fertile lands are hardly allocated for cassava cultivation. However, in the middle belt of Nigeria, inadequate rainfall and preference for other staple crops are the major challenges confronting cassava production. In the north, soil conditions favor extensive mechanized farming while rugged topography, rock outcrops, and tree trunks are hinderances to mechanized farming in the south.

The comparison of the influences of climate and soil on cassava production across Nigeria shows contrasting influences. For instance, the grouping of stations based on climatic conditions does not reflect the grouping based on soil conditions (Table 6). Our results conform to the multi-location/multi-demonstration trial (MLT/DEMO) and the on-farm trial (OFT) experimental results (Fig. 4). The highest yields were recorded in the Southern Guinea Savanna (45 t ha^−1^ in Mokwa in Niger State) and rainforest region (40 t ha^−1^ in Warri in Delta State) for MLT/DEMO and OFT, respectively. Therefore, optimum cassava yield performance requires moderate rainfall, moderately high temperature, moderately low altitude, high sunshine duration, and moderate- to high-soil fertility rate. Further, the cassava yields vary significantly with soil characteristics, even within the same A-C zone. As earlier established, crop yield performance largely depends on soil conditions where climatic condition is optimal^11^.

**Table 6:** Suitability scores of the on-farm locations using the summation method (C and E mean climatic and edaphic scores/conditions, respectively).

Group	Location Code	^a^C&E	Suitability Class Code	Suitability Class Code Definition (variations in *C* & *E* conditions)	Rating Scores		Suitability Scores
					*C*	*E*	*Total Score*
A	16	1,1	SZ1	best climatic and edaphic	15	19	34
B	6,13,14	3,1	SZII	third-best climatic but best edaphic	13	19	32
C	12	2,2	SZIII	second-best climate and edaphic	14	17	31
D	5	1,4	SZIV	best climatic but second least edaphic	15	14	29
E	9	2,4	SZV	second-best climatic but second least edaphic	14	14	28
F	10	4,3	SZVI	second-least climatic but third-best edaphic	12	15	27
G	1	3,4	SZVII	third-best climatic but second least edaphic	13	14	27
H	4,11	4,4	SZVIII	second-least climatic but edaphic	12	14	26
I	2,3,7,8	4,5	SZIX	second least climate but the least edaphic	12	11	23
J	17	5,4	SZX	least climate but second least edaphic	9	14	23
K	15	5,5	SZXI	least climate and edaphic	9	11	20

### On-farm cassava yield in Nigeria

**Figure 6:**
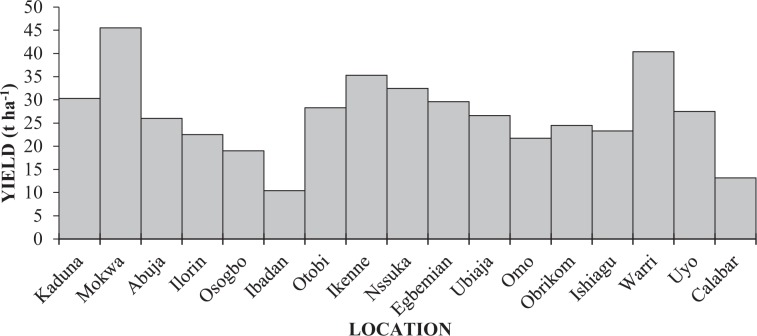
Quantitated Cassava yield performance (t ha^−1^) across the on-farm trial locations in Nigeria.

The average cassava yield (26.86 t ha^−1^) for the trial farms is higher than that recorded for Nigeria (10.18 t ha^−1^). In Fig. 6, we depict our findings on the on-farm trial yield in Nigeria. The values range from 10.4 t ha^−1^ in Ibadan (in the savanna of South-western Nigeria) to 45.5 t ha^−1^ in Mokwa (in the Guinea savanna in North-central Nigeria). In-between, the cassava yield is as high as 30.3 t ha^−1^ in Kaduna (in the Sudan savanna of North-western Nigeria) whereas, in the rainforest belt of Southern Nigeria, 40.4 and 35.3 t ha^−1^ were obtained in Warri and Ikenne, respectively. Interestingly, the cassava yields from16 out of 17 on-farm trial locations are higher than that of the global average (11.1t ha^−1^).

### Geospatial relationship between cassava yield and environmental parameters

**Figure 7:**
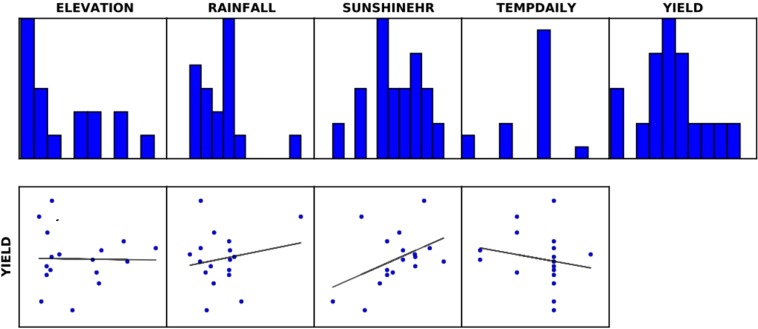
Distribution of the predictor variables and their relationships with cassava yield (the dependent variable).

Tables 7 and 8 present the variables and diagnostics of the ArcGIS-based Ordinary Least Square (OLS) analyses. Probability analysis shows that the coefficient is statistically significant for only the sunshine hour at α = 0.001 (Table 7). However, Koenker (BP) statistic (Table 9) indicates that all modeled relationships are consistent, both independent. The robust probabilities (Robust_Pr) reveals that cassava yield-sunshine hour and cassava yield-rainfall relationships are significant at α = 0.1 unlike cassava yield-temperature and cassava yield-elevation relationships. Further, Joint Wald statistics shows that the OLS model is reliable. Also, the variance inflation factor (VIF) indicates no redundancy among the explanatory variables. And, both the Jarque-Bera statistic and the multiple R-squared values (R^2^ = 0.50) indicate that the model is reliable sufficiently. Thus, all the model parameters are relevant to cassava yield prediction in the tropical environment. Nevertheless, an increase in sample size would enhance the performance of the regression model. Therefore, the adopted methodology is suitable for cassava yield prediction in the tropics. However, the model accounts for only 50% of the regression plain, indicating that there are other parameters of influence on cassava yield in Nigeria. Therefore, the OLS model professes daily sunshine hour and rainfall as the most important weather parameters to the cassava yield potential in Nigeria (Table 9 and Fig. 7). The Least Square models show that cassava yield exhibits a positive and significant association with the sunshine hour and rainfall. An inverse, but the insignificant relationship is also evident between cassava yield and daily temperature. Moreover, elevation does not portend any influence on the cassava yield.

**Table 7:** Result summary of the OLS model analysis amongst the variables.

Variable	Coefficient	Std. Error	t-stat.	Probability	Robust_se	Robust_t	Robust_pr	vif
Intercept	−0.7308	78.26	−0.0093	0.9927	53.14	−0.0138	0.9893	—
Elevation	−0.0073	0.0107	−0.6824	0.5080	0.0111	−0.6585	0.5226	1.3402
Rainfall	0.0102	0.0048	2.1003	0.0575	0.0038	2.693	0.0196*	1.9211
Sunshine hour	0.0134	0.0041	3.2922	0.0064*	0.0034	4.011	0.0017*	1.6175
Temp. daily	−0.5988	2.603	−0.2301	0.8220	1.690	−0.3543	0.7292	1.3007

**Table 8:** Ordinary Least Square (OLS) diagnosis parameters and yield values (*means parameter value is significant).

Input Features	Yield	Dependent variable	Yield
Number of observations	17.01	Akaike’s information criterion (AICc)	129.7
Multiple R-squared	0.5017	Adjusted R-squared [d]	0.3356
Joint F-statistic	3.021	Prob (>F), (4,12) degrees of freedom	0.0614
Joint Wald statistic	31.58	Prob (>chi-squared), (4) degrees of freedom	0.000002*
Koenker (BP) statistic	4.404	Prob (>chi-squared), (4) degrees of freedom	0.3541
Jarque-Bera statistic	1.802	Prob (>chi-squared), (2) degrees of freedom	0.4062

**Table 9:** Summary of linear regression model: cassava yield ≈ altitude + precipitation + sunshine hour + temperature [(Significant codes: 0 ‘***’ 0.001 ‘**’ 0.01 ‘*’ 0.05 ‘.’ 0.1 ‘’ 1) (Residual standard error: 7.174 on 12 degrees of freedom) (Multiple R-squared: 0.5017; Adjusted R-squared: 0.3356) (F-statistic: 3.021 on 4 and 12 DF, p-value: 0.0614) Cassava yield ~ sunshine hour is significant at α = 0.001, Cassava yield ~ rainfall is significant at α = 0.06)].

Parameter	Estimate	Std. Error	t value	Pr (>|t|)
Intercept	−0.7308	78.27	−0.009	0.9927
Altitude	−0.0073	0.0107	−0.682	0.5080
Rainfall	0.0102	0.0048	2.100	0.0575
Sunshine-hour	0.0134	0.0041	3.292	0.0064**
Temperature	−0.5988	2.6028	−0.230	0.8220

### Validation of the zoning map

Table 10 shows that all the trial farms are located either within the *suitable* (11.8%) or *very suitable* (88.2%) suitability zone. About 59%, 23.56%, and 17.76% of the trial farms had yields of range 26–30, 36–45, and 10–20 t ha^−1^, respectively. The disparity in cassava yields across the trial farms could be attributed to the difference in the periods the farmlands have been under on-farm management practices. Usually, continuous cassava cultivation on the same piece of land results in intense depletion of soil fertility and consequent reduction in yield quantity and quality^8,50^.

**Table 10:** Quantitated agreement between cassava yield and suitability rating.

Cassava yield range (t ha^−1^)							
Zones of trial farms	(10–15) %	(16–20) %	(21–25) %	(26–30) %	(31–35) %	(36–40) %	(41–45) %
Not Suitable zone	—	—	—	—	—	—	—
Very marginal zone	—	—	—	—	—	—	—
Marginal zone	—	—	—	—	—	—	—
Moderate zone	—	—	—	—	—	—	—
Suitable zone	—	—	—	11.8	—	—	—
Very Suitable zone	11.8	5.9	23.5	23.5	11.8	5.9	5.9
Most Suitable zone	—	—	—	—	—	—	—
Total	**11.8**	**5.9**	**23.5**	**35.3**	**11.8**	**5.9**	**5.9**

Within the *very suitable* zone, cassava yield ranged from 10 t ha^−1^ (at Ibadan where the trial farm has been under cultivation for over a decade) to 45 t ha^−1^ (at Mokwa where soil fertility of trial farm is very high), indicating the decisive influence of soil fertility status on cassava yield. The suitability rating predicts the yield potential of all the on-farm trial locations with high accuracy.

### Cassava production prospects in Nigeria

**Figure 8:**
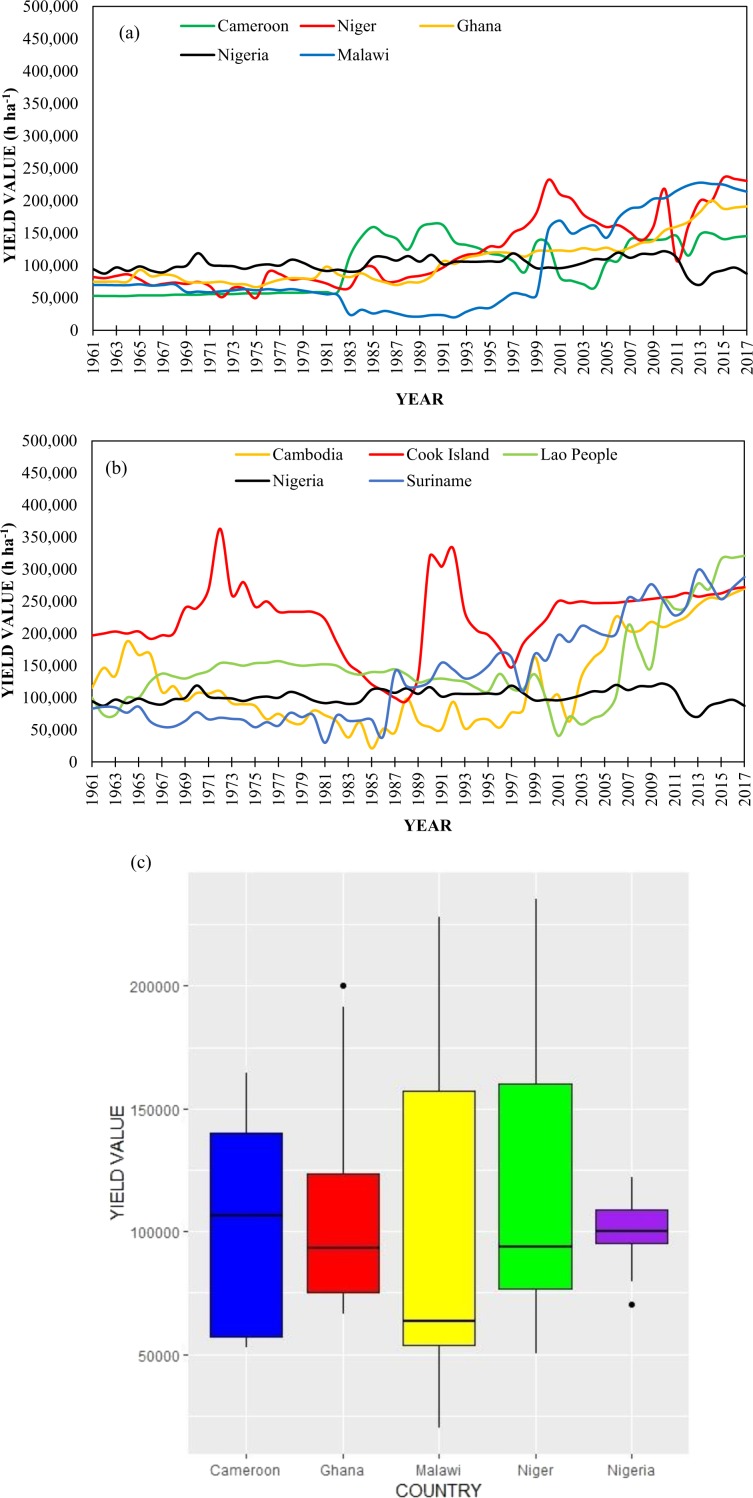
Comparison of cassava yield trend of Nigeria with countries with the highest yield per hectare in (**a**) Africa (**b**) the world. (**c**) Summary of cassava yield (h ha^-1^) data for the five African countries with the highest cassava yield between 1961 and 2017.

As of 2017, Nigeria was the global leading producer of cassava with 59,485,947 t, with 8.7 t ha^−1^ average yield^13^. To achieve that, Nigeria cultivated the sum of 6,792,349 ha of land, which equals 11.2% of the total suitable and cultivable lands in Nigeria. Figure 8a shows that not until 1982, cassava yield Nigeria was comparatively superior to other African nations’. While the global average cassava yield is 11.1 t ha^−1^ high, the value in Laos has tripled in recent times (Fig. 8b). For the 57-year period (i.e. 1961–2017) under investigation, the highest cassava yield recorded for Nigeria was 12.22 t ha^−1^, with the highest average in Africa between 1961 and 1979. However, between 1980 and 2010, the cassava yields fluctuated around 10 t ha^−1^ in Nigeria, and since 2011, it has been below 10 t ha^−1^, having the lowest yield in 2013 (Fig. 8b). As depicted in Fig. 6a, the cassava yield in Nigeria was higher than those of Laos, Suriname, and Cambodia (the three current record holders of highest cassava yield on the planet) from 1961 to 1980. Thus, the observed trends in those trios indicate that Nigeria has the innate potentials to rank top if more lands are cultivated and necessary technological means are adopted. Table 11 presents the descriptive statistics of cassava yield data for the five (5) leading African producing nations. Nigeria has the third highest mean yield (10.18 t ha^−1^), lower than Niger’s (11.98 t ha^−1^) and Ghana’s (10.69 t ha^−1^). Albeit, Nigeria has the lowest maximum yield (12.22 t ha^−1^) and the highest minimum yield (7.03 t ha^−1^) while the highest maximum yield (23.53 t ha^−1^) and lowest minimum yield (2.01 t ha^−1^) were recorded for Niger and Malawi, respectively. Figure 8c provides summarized cassava yield stock chat for Fig. 8b.

**Table 11:** Descriptive statistics of cassava yield (h ha^−1^) for five leading producers of cassava in Africa (^a^Q1 denotes 25 percentiles; ^b^Q3 denotes 75 percentiles).

Country	Mean	Median	Maximum	Minimum	^a^Q1	^b^Q3
Cameroon	100015	106556	164386	52991	56000	140000
Niger	119839	94000	235354	50312	75000	160000
Ghana	106935	93333	200239	66667	75000	125000
Nigeria	101796	100000	122155	70323	93800	108000
Malawi	94898	63500	228041	20140	53000	158500

The h in h ha^−1^ means hectogram (1 hectogram = 0.0001 ton).

About 52.6% of Nigeria’s yield (for the 57-year study period) is between the range of 9.38 and 10.8 t ha^−1^ while 26.3% is within 10.9 and 12.22 t ha^−1^ and the remaining 21.1% between 7.03 and 9.36 t ha^−1^. These indicate a consistently low cassava yield in Nigeria. Further, Fig. 6c shows a skew down in yield in Malawi, Nigeria, Niger, and Ghana albeit a skew up in Cameroon. Excessively low (7.03 t ha^−1^) and excessively high (20.02 t ha^−1^) outliers were recorded for Nigeria in 2013 (attributed to the impact of infectious cassava diseases^2,51^) and Ghana (owing to the increased focus on cassava cultivation^29^) in 2014, respectively.

### Projections of cassava production based on likely climate change impacts

**Figure 9:**
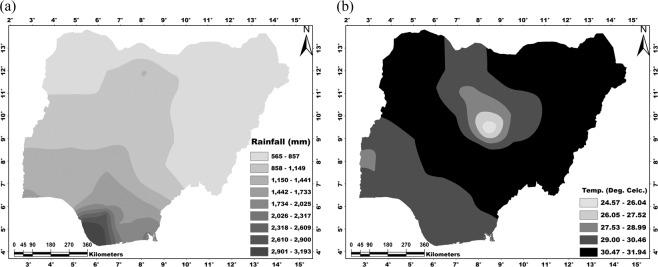
Projected (**a**) annual rainfall amount (2070–2099) based on 7.5% decrease^18^ and (**b**) daily average temperature (2046–2100) based on 0–4 °C increase^22,62^. Maps were generated using ArcGIS 10.5 software.

Based on the annual rainfall variation (Fig. 2a), our projections indicate 565 mm in the north to 3,193 mm on the coast average annual rainfall during 2070–2099 climatic age^18^ (Fig. 9a). On the other hand, the projected average daily temperature (based on Fig. 2b) for 2046–2100 (Fig. 9b) shows a range of 24.57–31.94 °C, especially in the northeastern, northwestern the southern central parts of Nigeria^22^. Considering the tolerance range of cassava for temperature and rainfall in Nigeria, more suitable farmlands will be available for cassava production in the future, particularly in the current cassava production belt. The spatial patterns of future temperature and rainfall indicate possible changes in future cropping systems in Nigeria, i.e., cultivation interest of tree crops (such as cocoa and cola nut) will likely be compromised for stable crops (such as rice, cassava, yam, and cocoyam) particularly in southern Nigeria. Also, the predicted increase in rainfall in the Northern Nigeria^18^ also indicates that areas currently unsuitable for cassava production (due to inadequate rainfall) could become suitable^18^. Conclusively, the future climate will favor stable crop production in Nigeria, although at the expense of cash crop production in the south.

### Discussion

The yield performance of a given crop largely depends on the climate, soil conditions and on-farm management practice^3,5,8,11,15,16^. From our study of the 17 trial farms, soil characteristics account for 73% of suitability scores where the climate was less favorable for cassava cultivation. Similarly, the climatic conditions account for 82% of the suitability scores where soil characteristics were less favorable for cassava cultivation. At Mokwa, Central Nigeria, both the climatic parameters and the soil resources are pertinent to achieving an average cassava yield above the world average (11.1t ha^−1^). With our cassava clone (TMS98/0505), any location with optimal soil conditions and favorable climatic conditions (characterized by the long daily sunshine hour) is favorable^3^. By observing the yield performance and suitability rating of the locations (Figs. 4 and 5), we realized that the TMS98/0505 clone would strive excellently in the Northern Guinea Savanna, a region popularly tagged *unsuitable* for cassava cultivation^14,15^. Specifically, the high yield recorded in Kaduna (where environmental conditions are barely favorable)^14,15^ indicates that cassava growth requires *moderately favorable* climatic conditions to thrive appreciably in Nigeria. Therefore, to enhancing food security in Nigeria, the annual cassava production capacity can be increased by extending the cultivation to other regions fairly favorable for the root crop. That the favorable zones are concentrated in the dry rainforest and southern Guinea savanna suggests that optimum cassava production requires the following conditions: well-drained sandy-loamy/loamy soils, evenly distributed but slightly high annual rainfall amount, moderately high temperature, and long sunshine hour^3,29^.

The combined influence of climate and soil characteristics on crop production in sub-Sahara Africa has been appraised^6,9,11,14,15,17,18^. Our results suggest that climatic conditions exert the most limiting effects on cassava production in the semi-arid region of Nigeria. On the other hand, the dissected topography and the natural forest constitute a major barrier against mechanize farming in the south. The current climatic regime poses a great threat to the widely practiced rainfed agriculture in Nigeria^7,9,17^. Also, the increasing number and severity of drought events are hindering crop yield and consequent food security in Nigeria^14,15^. However, Nigeria could take advantage of her abundant renewable water resources to compensate for soil moisture deficits particularly in the semi-arid region. This could be achieved by retaining a large percentage of the annual runoff for irrigation purposes during the dry season. On another note, one of the strategies to maximizing the productivity of land resources is sustainable land allocation^38,47^. Thus, our generated suitability map (Fig. 4) could serve as a decision-making tool for cassava production intervention in Nigeria, especially now that the country is prioritizing the agricultural sector to diversify its economy. So far, inadequate rainfall and short planting period are the major factors inhibiting cassava production in Northern Nigeria; a region characterized by the extended sunshine hour. Thus, cassava farming would strive where viable irrigation scheme is available in Northern Nigeria. Moreover, the observed variability in edaphic suitability proffers that soil-related constraints are field-specific, so also the required intervention^3,52,53^. Therefore, a need to revolutionize farming system in Nigeria is sacrosanct. Contrary to the assumption that cassava strives optimally on nutrient-depleted soils^54–56^, the highest yield was recorded for a trial farm located in Mokwa, Central Nigeria where climate and edaphic conditions are highly suitable.

In future Nigeria, there will be a general increase in temperature and a decreased annual rainfall in the southern and central regions^21–23,56^. These predictions indicate that tree crops will likely be jettisoned for annual (staple) crops (such as cassava) that have a wider tolerance for extreme temperature and low moisture availability. Thus, cassava has the potential to become an essential crop for Nigeria in the future. However, there is an urgent need for a paradigm shift from traditional (crude) farming practice to intensive agricultural practice^57^.

The positive impacts of effective on-farm management, soil fertility enhancement (organic and inorganic fertilizer applications) and the allocation of suitable lands on cassava yields have been reported in some Sub-Sahara African countries^5,24–27,30,58^. One of the major factors responsible for increased cassava yield in Niger, Malawi, Ghana, and Cameroon is effective on-farm management such as mulching and soil tillage^29^. Other factors accounting for increased yield in these countries include the dedication of suitable (fertile) land to cassava cultivation and the use of fertilizer where required^52,53,59–61^. However, in Nigeria, the reverse is the case, i.e., cassava is usually cultivated on nutrient-depleted soil. Also, farmlands across the major planting belt lack the required on-farm management practices. Nigerian farmers also bear the mentality that fertilizer application to cassava farm is unnecessary and wasteful^2^. From our regression modeling, the relevance of other environmental variables that influence cassava yield in Nigeria was confirmed, i.e., aside from the climate, soil conditions are key to cassava yield^31,58^. Our field investigations showed that farmers are still cultivating the local varieties of cassava even in South-western Nigeria where IITA is located. All the aforementioned reasons collectively contribute to low cassava yield in Nigeria^1^. Therefore, efficient on-farm management practices, fertilizer application, and farmers’ accessibility would improve cassava yield in Nigeria.

Finally, the large difference in yield (18.16 t ha^−1^) between the average yields of trial farms and and those of smallholder farmers is confirmed as a major challenge that must be overcome in the sub-Sahara Africa^3^. The relegation of cassava to marginal and nutrient-depleted land has contributed to its low yield in Nigeria. We estimate that if Nigeria improves on the accessibility of farmers to improved varieties, current farm management system and soil fertility enhancement of the farmlands for cassava production, the nation can produce more than 120,000,000 t of cassava per annum. Consequently, in contrast to the qualitative methods previously deployed for A-C zoning in Nigeria^14,15^, our method permits multi-criteria analysis that enables the integration of multiple environmental variables. It also allows for permanent isolation of unavailable lands considered as part of suitable zones.

### Conclusion and future work

Currently, the cassava yield in Nigeria is lower than those of some African countries such as Niger, Ghana, Malawi, and Cameroon (FAOSTAT, 2019). For in-depth investigation and future projections, we delineated Nigeria into various climato-edaphic homogenous zones for cassava production on the GIS environment. We discovered that soil conditions exert more influence on cassava yield performance than climatic conditions in Nigeria. The climatic parameters that influence cassava yield decisively are the sunshine hour and rainfall. Favorable cultivation zones are mostly found in the dry rainforest and southern Guinea savanna (the two major cassava production belts in Nigeria). However, cassava could be profitably cultivated in the northern marginal zones where alternative water source (e.g. viable irrigation scheme) is available. Also, on-farm cassava yields are higher than the modeled values, suggesting that Nigeria has a high potential to improve on the current cassava yield. Because the estimated yield data provided by FAOSTAT might not be a true representation of cassava yield performance in Nigeria, a nation-wide crop yield data gathering and archiving is required. The selected cassava cultivar (TMS98/0505) performed optimally even in regions where climatic conditions are marginal. Even with our future projections (considering likely future climatic variabilities with temperature and rainfall), the cultivar will strive excellently if other A-E and A-C factors aforementioned are satisfied. Hence, future cassava production interventions in Nigeria should place emphasis on soil conditions.

Although we have demonstrated that the acquired data are adequate to predict cassava yield in Nigeria, still, there is a need for further research on the *in situ* influence of soil fertility on cassava yield. Also, it is imperative to study the yield performance of cassava cultivars during seasonal rainfall and under irrigation schemes. Moreover, more research on farmers’ accessibility to newly cloned crop varieties in Nigeria is exigent. Likewise, it is imperative to study the spatio-temporal changes in regional suitability for cassava cultivation, especially under various climate change scenarios in Sub-Sahara Africa. Finally, the existing focus of cassava breeders on cloning of the cultivars resistant to diseases (such as mosaic and blight diseases)^2^ should also be replicated toward the production of cultivars tolerant of drought and extreme temperatures.

## Supplementary information

Supplementary Information.
