# Supplementary material for: Agro-Climato-Edaphic Zonation of Nigeria for a Cassava Cultivar using GIS-Based Analysis of Data from 1961 to 2017
Source: Sci Rep. 2020 Jan 27;10:1259. doi: 10.1038/s41598-020-58280-4 (PMC6985172; doi:10.1038/s41598-020-58280-4)
Supplement: Supplementary file 1 — Supplementary Information. [file 41598_2020_58280_MOESM1_ESM.pdf]

SUPPLEMENTARY INFORMATION FOR FIGURE 5 (SI 1)

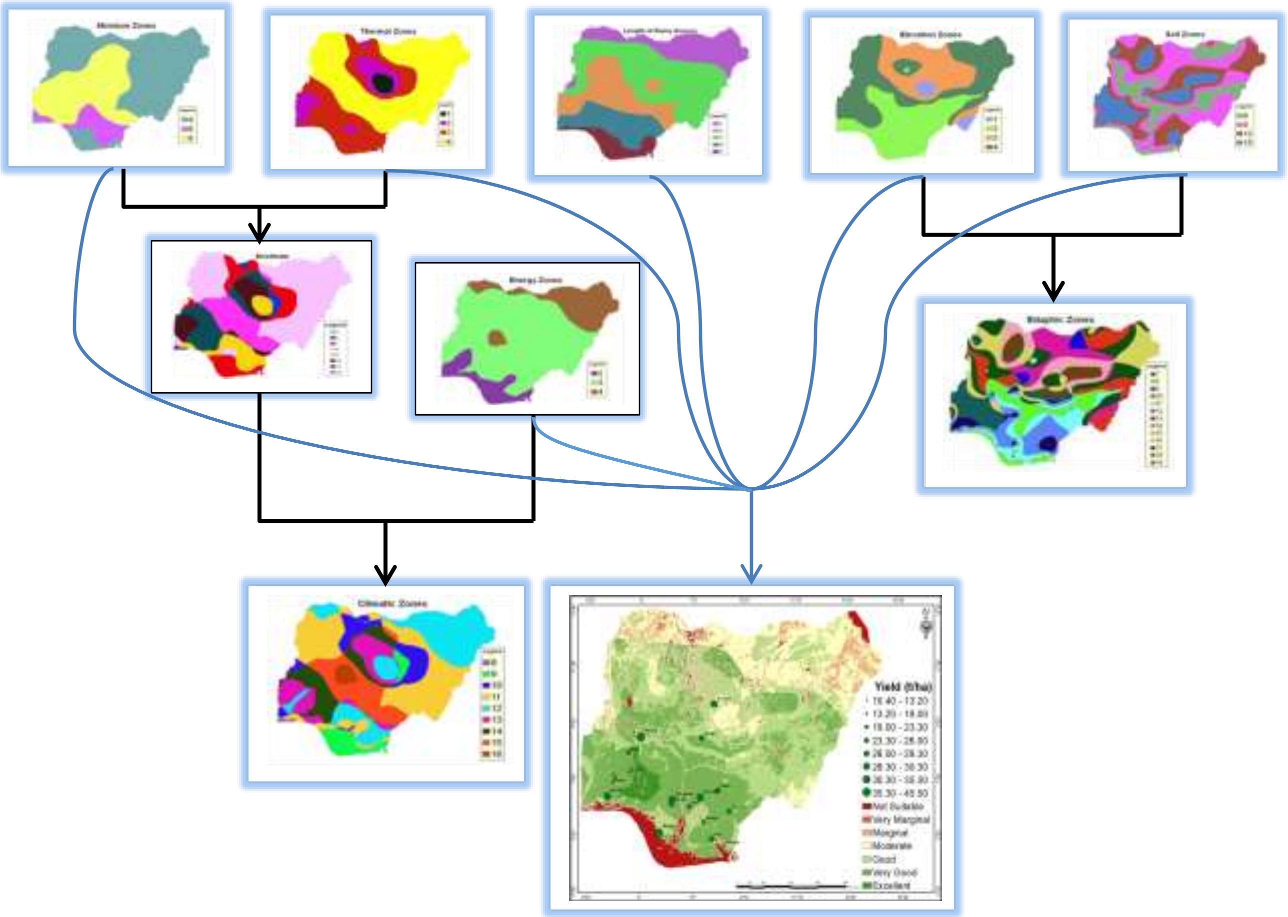

SI 1. Input raster files, climatic zones, edaphic zones and the final agro-ecological zone map for cassava production (Refer to **Table 1**).

Table 1. Reclassified input parameters and their internal ratings.

	Rainfall			Temperature			Sunshine Hour			Altitude			Duration of Wet Season			Edaphic factor		
Range	5000-2001	2000-1000	999-500	40-29	28-25	24-11	10-8	7-5	4-3	1500-360	359-150	149-100	10-9	8-7	6-5	15-13	12-10	9-7
Class	3	1	2	3	1	2	1	2	3	1	2	3	1	2	3	1	2	3
Scale Value	7	9	8	5	9	8	9	8	7	9	8	7	9	8	7	9	8	7

SUPPLEMENTARY INFORMATION FOR FIGURE 4 (SI 2)

Soil Suitability

Class I – There is no class I soil (i.e. very good land with minor or no physical limitations to mechanical cultivation)

Class II Soils – Fairly Highly Suitability (SI)

In this class are moderate to a good land with few physical limitations to mechanized cultivation but which can be corrected. This class will include mapping units I and II. These soils are found on nearly level to gently undulating plains with slope ranges of 0 – 2% and are derived from sandstone, shale and basement complex materials. The soils are deep, well-drained. They have loamy sandy to sandy loamy surfaces underlay by sandy clay loam to sandy clay subsoil. However, some soil units have a limitation due to poor internal drainage, resulting in excessive wetness. The texture is also heavy due to the underlying parent material – basalts. Class II soils cover 27.51% of Nigeria. The major crops grown on these soils include guinea corn, maize, and vegetables.

Class IIs Soils – Moderately Suitable (S2)

In this class are moderately suitable lands with limitations such as moderate to shallow depth due to the presence of rock outcrops and inselbergs. This class includes mapping unit III. The soils occur on gently undulating to undulating plains with scattered rock outcrops and inselbergs. The slope ranges are 2 – 6%; while the soils are derived from sandstone, shales and undifferentiated basement complex. The soils are shallow to moderately deep and deep, well-drained. They have sands to sandy loam surfaces underlain by sandy clay loam to sandy clay subsoils. The soils of this class cover 17.38% of the country's landmass.

Class IIIe,s Soils – Marginally Suitable (S3)

In this class are fair to good lands that can be mechanically farmed with great care. There are moderate to high erosion hazards owing to undulating hilly dissected topography with scattered rock outcrops. Other limitations include shallow depth to the iron pan, concretions or stony/gravelly substratum. This class includes mapping unit IV. The soils are found on undulating dissected plains with scattered rock outcrops and hills. The slope ranges are 6 – 13% while the soils are derived from sandstone and undifferentiated basement complex and ironstone sheets. The soils are shallow to moderately deep and well-drained. They have sand to loamy sand surfaces underlain by loamy sand, sandy loam to sandy clay loam subsoil. The soils of this class cover14.03% of Nigeria's landmass.

Class IVe,s Soils – Currently Not Suitable (N1)

In this class are lands not suitable for mechanical cultivation but suited for limited clearing and land cultivation for the production of perennial crops. The soils commonly occur on hilly or steep topography. Soils are better used for forest reserve and wildlife. The limitations include moderate to severe erosion hazards, steep or rocky slopes, very shallow depth, and stoniness. Other limitations are low permeability, low moisture-holding capacity,

and poor capacity to utilize added fertilizer. The establishment of stringent erosion control practices is essential even when such soils are hand cultivated. This class includes mapping unit V. the shallow soils occur on dissected plateau plains, hills and ridges. The slope ranges are 13 – 55% while the soils are derived from sandstone, shale and undifferentiated basement complex. The soils are shallow and well-drained. They have sand to loamy sand surfaces underlain by bedrock. The soils of this class cover 27.51% of Nigeria's landmass.
